# Developing and implementing a self-monitoring toolkit for a coordinated multinational randomized acupuncture trial

**DOI:** 10.1186/s12906-022-03648-4

**Published:** 2022-06-17

**Authors:** Sylvia Baedorf Kassis, Weidong Lu, Sarah A. White, Im Hee Shin, Sung Hwan Park, Young Ju Jeong, Chang Yao, Jennifer Ligibel, Barbara E. Bierer

**Affiliations:** 1 Multi-Regional Clinical Trials Center of Brigham and Women’s Hospital and Harvard, Cambridge, MA USA; 2 Division of Global Health Equity, Department of Medicine, Brigham and Women’s Hospital, Boston, MA USA; 3 Department of Medical Oncology, Dana-Farber Cancer Institute, Boston, MA USA; 4 Department of Medical Statistics & Informatics, School of Medicine, Daegu Catholic University, Daegu, Republic of Korea; 5 Department of Surgery, School of Medicine, Daegu Catholic University, Daegu, Republic of Korea; 6 Department of Oncology, Jiangsu Provincial Hospital of Traditional Chinese Medicine, Nanjing, China; 7 Department of Medicine, Brigham and Women’s Hospital, Harvard Medical School, Boston, MA USA

**Keywords:** Performance monitoring, Regulatory binder, Self-assessment, Quality assurance, Clinical trial

## Abstract

**Background:**

In 2019, investigators from China, South Korea and the United States of America initiated a coordinated multinational trial. The trial included three parallel randomized studies with a planned pooled analysis of individual patient data, to test the effectiveness of acupuncture on hot flash-related symptoms in hormone receptor-positive breast cancer patients prescribed adjuvant endocrine therapy. Given the study's approach, there was no central coordinating center or data monitoring committee for the study, so a site performance self-monitoring toolkit was developed and implemented to support study teams in collecting and maintaining high-quality regulatory information, and consistent review of study data and documentation.

**Methods:**

The site performance self-monitoring toolkit was created based on best practices related to post-approval quality assurance/quality improvement (QA/QI) procedures that support data quality. The toolkit included: (1) a binder of essential study management documents and related monitoring logs for sites to complete and maintain (herein called regulator binder), (2) a study start-up checklist, (3) a self-assessment study conduct and oversight checklist to be completed regularly, and (4) a study close-out checklist. In addition, a process of regular virtual meetings to discuss documentation progress coupled with periodic external remote review of completed logs and checklists provided accountability checks.

**Results:**

Over the course of the study, the sites in China and South Korea completed the entirety of the site performance self-monitoring toolkit, and successfully submitted their completed materials for review. The process of implementing a self-monitoring toolkit in a multinational integrative medicine study is described qualitatively. Periodic external review of the completed toolkit materials revealed categories of findings. Written follow-up reports were provided to sites and discussion of the documents occurred via separate virtual meetings.

**Conclusions:**

Site study team self-monitoring provides a feasible, consistent, and effective way to review the collection and maintenance of data and regulatory documentation for quality assessment in minimal risk clinical research studies and can augment formal study monitoring activities in higher risk studies. Iterative feedback and support appeared to drive a disciplined approach to maintaining regulatory document compliance and helped sustain investigator and study team engagement in the process.

**Trial registration:**

ClinicalTrials.gov Identifier NCT03783546 (21/12/2018).

## Background

Clinical trial monitoring entails systematic activities to ensure that studies are conducted and data are acquired and documented according to the planned and approved protocol in compliance with the International Council for Harmonisation of Technical Requirements for Pharmaceuticals for Human Use (ICH) Good Clinical Practice (GCP) E6 (R2) guidelines and relevant regulations [1]. Specifically, the addendum to Sect. 5 of ICH GCP E6(R2) states that a "systematic, prioritized, risk-based approach to monitoring clinical trials" (pg. 29) should be followed. Clinical trial monitoring requires routine reviews of study documents and protocol adherence. While monitoring is frequently accomplished by a professional, designated committee, or organization external to the study team, there is an important role for self-monitoring by local study team members. Depending on the risk profile of the study, self-monitoring can provide meaningful insight that helps the study team confirm its adherence to the protocol, regulations, and concordance with ICH GCP.

The research purpose, strategy, and procedures in clinical studies involving integrative medicine are frequently different from those in trials of pharmacological interventions conducted for the purpose of gaining regulatory approval to market a new drug entity [2]. Nonetheless, the protection of human participants, overseeing the research steps in the study plan, and confirming the accuracy of the data collected are equally important in clinical trials of integrative medicines as in drug and device studies [2].

In 2019, investigators from China, South Korea, and the USA initiated a coordinated multinational trial, including three parallel randomized studies with a planned pooled analysis of individual patient data, to test the effectiveness of acupuncture on hot flash-related symptoms in hormone receptor-positive breast cancer patients prescribed adjuvant endocrine therapy. Given that randomized trials of integrative therapies rarely enroll patients from more than one country, this study was an innovative opportunity to obtain comparative data from participants in three countries. The study design and protocol will be described separately. The trial is registered at ClinicalTrials.gov (ClinicalTrials.gov Identifier NCT03783546, 21/12/2018).

The trial was planned as separate parallel studies using an agreed-upon common protocol implemented across the three countries. There was, therefore, no centralized coordinating center or data safety monitoring board/data monitoring committee. Monitoring was nonetheless recognized by the study teams as an important requirement of clinical research that must be planned prospectively, carried out consistently, and documented. Here we provide a qualitative description of clinical trial self-monitoring strategies including a process of remote review and feedback.

## Methods

The trial's monitoring plan was designed to (1) provide a toolkit that promotes and facilitates self-monitoring, and (2) support the sites by conducting remote review of the various toolkit components including the study's essential study management documents.

Members of the Multi-Regional Clinical Trials Center of Brigham and Women's Hospital and Harvard (MRCT Center) were involved as external advisors to the study teams in their completion of the site performance monitoring toolkit. The self-review was predicated upon a site performance self-monitoring toolkit that was developed and distributed to the sites to support the collection and maintenance of high-quality regulatory information and other related study documentation. The study relied on a robust process of regular self-assessment and intermittent remote review and feedback from the MRCT Center team. A description of the toolkit, including essential study management documents, logs and checklists, and the remote oversight process follows.

The site performance self-monitoring toolkit was created based on best practices related to post-approval quality assurance/quality improvement (QA/QI) procedures to ensure data quality [3]. The site performance monitoring toolkit and process included the following:

- ○ An adapted study-specific binder of essential study management documents (the regulatory binder) and associated tabs for document storage and maintenance [4].
- ○ A library of template study logs to support completion of the regulatory binder.
- ○ A study start-up checklist to support study initiation.
- ○ A self-assessment checklist to be completed by study team members at regular intervals throughout the study.
- ○ A study close-out checklist to be completed once data analysis was complete and then again later when the study was closed.
- ○ Regular review throughout the study of completed essential study management documents of the regulatory documents and checklists.
- ○ Feedback process with written reports and discussion at monthly virtual meetings and intermittent individual virtual site check-in meetings.

Because of unanticipated changes to study activities caused by the onset of the COVID-19 pandemic at the beginning of 2020, a few tools were updated or developed anew to reflect pandemic-related issues. For example, sites were provided with an updated study deviation log and adverse event log to capture pandemic-related issues, plus a new unanticipated problems log. Additional information about the components of the toolkit is included in Table 1.

**Table 1:** Description of the tools and components of the site performance self-monitoring toolkit

**Tool**	**Description and components**
**Regulatory binder**	A template and guidance document for tracking essential study management documentation associated with the hot flash study. It was designed to help study sites achieve and maintain regulatory compliance and adhere to common standards of practice in the conduct of research involving human subjects Components: The sections of the regulatory binder were tailored to the planned acupuncture trial and included: •Protocol •Curriculum Vitae (CV) •Licensure •Study Logs oEthics Committee Communication Log oPre-Screening Log oEnrollment Log oDelegation of Responsibility Log/ Staff Signature Log oStudy monitoring Log oAdverse Event Tracking Log (updated during pandemic) oAdverse Event Reporting Form oProtocol Deviation/Exception Tracking Log (updated during pandemic) oTraining Log oUnanticipated Problem Log (newly added during pandemic) •Ethics Committee (EC) •Consent Forms •Acupuncture Protocol •Data Collection •Sponsor •Training •Scientific Review •Other
**Study start-up checklist**	A checklist for investigators/study teams to review all tasks that need to be accomplished before the site is ready to initiate participant recruitment. For example, sites must ensure the appropriate approvals have been received, staff training has been fulfilled, and the site has created data collection forms. In order to ensure site readiness, a checklist of site-specific tasks and documents was created. The study start‐up checklist was tailored to a US site and our collaborators conducted a similar process as described above to modify sections to address requirements for site readiness in China and South Korea
**Self-assessment checklist**	A checklist for investigators/study teams to periodically evaluate their own compliance with reporting requirements and protocol compliance over the course of the study
**Study close-out checklist**	A checklist for investigators/study teams to use as a self-monitoring process at end of study enrollment and once the study is ready for final close-out

A representative of the MRCT Center joined the study launch meeting and each monthly virtual study meeting to solicit and discuss questions related to study documentation and data collection that may have arisen over the course of the study. Approximately twice a year, the study logs were reviewed for completeness and conformance with study expectations. Source documents were not reviewed for reasons of challenges in cross-border data transfer, anonymization of records, and translation. Further, it should be noted that these reviews were meant as a quality check of some key administrative research records and were not considered to be an audit of the study. Instead, this effort was intended to support accurate study documentation and inform concordance of conduct. As such, any observations were documented and discussed with the sites. Had critical issues been identified, arrangements to review additional study documents, including relevant source materials, would have been made.

## Results

Throughout the course of the study, the sites in China and South Korea regularly completed the toolkit components and submitted their completed checklists and logs for review by the MRCT Center. The resulting feedback process consisted of:

- Post-review written reports of observations that were provided to study teams, and included recommendations for how to address.
- Monthly all-site virtual meetings where all materials were discussed with the study teams on regularly scheduled three-country conference calls, as were any general findings from the review of site regulatory documentation procedures and self-monitoring activities. Discussion points included clarifying inconsistencies in documentation, soliciting questions that may have arisen in the sites' completion of the logs and checklists, and reviewing study expectations.
- Separate individual country virtual meetings to review materials and site-specific feedback with each of the sites. Individual discussions offered an opportunity to respond to any questions study teams had after reviewing the written feedback.

Specifically, the written reports shared with the study teams described areas where attention may have been needed to ensure that the associated logs, checklists, and other study documents were accurate and complete. Monitoring observations were grouped into categories for discussion and analysis (Table 2). No major deviations, unanticipated problems, or significant issues in documentation were identified.

**Table 2:** Findings and results—categories and select examples of review observations

**Category of Finding**	**Select Examples of Observations**
Administrative/oversight	-Internal monitoring/oversight activities should be documented on the study monitoring log (including self-monitoring) -Delegation log should be updated periodically and reviewed for timeliness and completion -Any errors in documentation should be crossed out with a single line and initialed
Omission	-The name of the person obtaining consent should be included on the enrollment log -The column indicating whether a person was given copy of consent should be complete -All entries on pre-screening log should be completed so that enrollments can be reconciled on enrollment log
Error	-Dates should follow the agreed upon convention (Month, Day, Year) -Log Page numbering should be entered and checked for accuracy -Staff on delegation log should correspond to staff study amendments to IRB/REC
Clarification needed	-The study staff end date should not be a date in the future but should be completed in real time -The types of training listed on the training log should be clarified
AEs and deviations	-Enrollment should have ceased while study approval/funding lapsed

## Discussion

Study performance and data quality monitoring is a critical part of the conduct of ethical and sound clinical trials. The site performance self-monitoring toolkit and remote monitoring activities were planned and performed as a QA/QI exercise. Like high-risk interventional trials, lower risk studies also require and can benefit from careful review of essential study management documents, helping to ensure compliance, data quality, and that study findings can be trusted. Serious documentation issues can be identified and discussed with the study investigators and their staff during the study, when correction for future performance is possible. Further, serious unanticipated problems, were they to occur, can be found early, reported to the local ethics committee, and mitigated. Regular self-review processes can identify potential issues in study conduct, processes, and documentation *early* in the study and, as a result, allow study teams to correct their processes, engage in education, or introduce new quality improvement efforts where needed.

There were several challenges in the implementation of the site performance self-monitoring toolkit and remote review process for this multinational clinical trial. The first related to language and translation. While investigators and study team members were able to read and speak English at all sites, translation of the documents into Chinese and Korean would have been important were the study to be scaled to additional multi-national performance sites. Second, coordinating virtual meetings across time zones was logistically challenging and could not be accomplished during typical working hours. Meeting times were not convenient for all participants, and success depended upon flexibility, motivation, and commitment to successful study completion. Third, selecting a user-friendly virtual conferencing tool that could be simultaneously used by all study teams was challenging. Due to different local regulations in each country, access to certain conferencing tools was limited. Finally, developing and implementing a suitable remote monitoring plan congruent with the risk and intensity of the clinical trial required balancing effort and time to completion with the likelihood of finding significant unanticipated problems. The resulting toolkit and schedule of meetings were well-received, and the sites completed all components of the toolkit. As previously noted, however, this remote monitoring activity was limited by both the lack of access to study source documents and the narrow scope of the external review process which was not intended to be formalized trial monitoring/auditing. While the risks of this trial were modest, a higher risk or more complex study might benefit from including in-person authentication and electronic submission and translation of source documents.

It should also be acknowledged that this study took place during the global COVID -19 pandemic and this a priori self-monitoring plan was fortuitous as the resulting global travel restrictions would have otherwise impacted any planned in-person monitoring activities. Here, however, monitoring was not affected. In fact, this approach allows for dynamic monitoring that is responsive to intercurrent and unpredicted changes to the research and the research environment. In addition, the approach is cost-effective and, in multinational studies, is time- and resource-efficient for monitors. At a minimum, all studies should consider implementing a site performance monitoring toolkit. While self-monitoring can be implemented in all studies, including minimal risk research and greater than minimal risk research, appropriate data and safety monitoring plans need to be developed for each individual study. As such, self-monitoring is a feature of a data monitoring plan but is often insufficient for research that is higher risk. Self-monitoring would not replace other independent measures in high risk research and would be complementary to and augment a formal data monitoring committee or external monitors in those instances.

## Conclusion

All interventional studies require a monitoring plan to help ensure ethical research conduct, regulatory compliance, and collection of quality data. This study utilized a site performance self-monitoring toolkit that included a series of logs and checklists to support study conduct through self-monitoring activities and remote monitoring whereby completed toolkit logs and checklists were reviewed by an external team. The monitoring plan described here was developed for this multinational, multi-lingual study at this level of complexity and risk, and was particularly appropriate given the emergence of a global pandemic. Self-monitoring and assessment are important components of study-specific monitoring plans.

## Data Availability

Site performance self-monitoring templates can be requested at mrct@bwh.harvard.edu.
